# Artificial eyespots on cattle reduce predation by large carnivores

**DOI:** 10.1038/s42003-020-01156-0

**Published:** 2020-08-07

**Authors:** Cameron Radford, John Weldon McNutt, Tracey Rogers, Ben Maslen, Neil Jordan

**Affiliations:** 1 Centre for Ecosystem Science, School of Biological, Earth and Environmental Sciences, University of New South Wales (UNSW), Sydney, NSW 2052 Australia; 2 Botswana Predator Conservation, Private Bag 13, Maun, Botswana; 3 Ecology and Evolution Research Centre, School of Biological, Earth and Environmental Sciences, University of New South Wales (UNSW), Sydney, NSW 2052 Australia; 4 Mark Wainwright Analytical Centre, University of New South Wales (UNSW), Sydney, NSW 2052 Australia; 5 Taronga Institute of Science and Learning, Taronga Western Plains Zoo, Taronga Conservation Society, Dubbo, NSW 2830 Australia

**Keywords:** Conservation biology, Behavioural ecology

## Abstract

Eyespots evolved independently in many taxa as anti-predator signals. There remains debate regarding whether eyespots function as diversion targets, predator mimics, conspicuous startling signals, deceptive detection, or a combination. Although eye patterns and gaze modify human behaviour, anti-predator eyespots do not occur naturally in contemporary mammals. Here we show that eyespots painted on cattle rumps were associated with reduced attacks by ambush carnivores (lions and leopards). Cattle painted with eyespots were significantly more likely to survive than were cross-marked and unmarked cattle, despite all treatment groups being similarly exposed to predation risk. While higher survival of eyespot-painted cattle supports the detection hypothesis, increased survival of cross-marked cattle suggests an effect of novel and conspicuous marks more generally. To our knowledge, this is the first time eyespots have been shown to deter large mammalian predators. Applying artificial marks to high-value livestock may therefore represent a cost-effective tool to reduce livestock predation.

## Abstract (web summary)

Radford et al. monitored cattle marked with artificial eyespots in non-commercial cattle farms in northern Botswana, and demonstrate that cattle with these eyespot markings were more likely to avoid predation by large carnivores. Their findings have applications as a cost-effective tool to reduce livestock predation by ambush predators.

## Introduction

Animals have evolved numerous strategies to avoid being eaten. Visual signals may reduce predation risk through camouflage, warning colouration, divergence and mimicry^1^. A classic example of anti-predator markings are eyespots on moth and butterfly wings^2–5^, but many other animal groups including other insects, fishes, molluscs, amphibians and birds, use concentric circles to deter predators^1,2,6–8^. While these patterns ostensibly resemble a vertebrate eye, the mechanism behind their anti-predator effects remains debated^7–11^. Some research suggests that eyespots simply act as deflection targets to divert attacks to non-vital body regions (the “deflection hypothesis”)^2,12–18^, while others suggest that eyespots are intimidating, either by mimicking the eyes of the natural predators of would-be attackers (the “predator-mimic hypothesis”)^2,8,19–23^, or alternatively by representing a novel or rarely encountered conspicuous feature that could promote avoidance behaviour (the “conspicuousness hypothesis”)^24–29^. Other studies have highlighted the importance of conspicuous eye-like signals occurring in pairs to be effective as anti-predator signals^29–31^. A further possibility, which has been suggested for some raptors^32,33^, is that eyespots may deceive predators or ‘mobbers’ into perceiving they have been detected, thereby preventing an attack (the “detection hypothesis”). These ‘pursuit-deterrent’ signals cause predators to re-evaluate the hunt based on the costs associated with the signal. In this case, eyespots may mimic prey vigilance, discouraging attack^34,35^. Conceivably, eyespots may have multiple anti-predator mechanisms, depending on the attacker, which are not necessarily mutually exclusive.

Although no known contemporary mammals display anti-predator eyespots, the effects of eye patterns and gaze have been shown to modify behaviour in this Class including in humans^36–38^, domestic and wild canids^39–41^, and domestic cats^42^. For example, eye images have been shown to increase human charitable donations in shopping malls^36^ and to decrease bike theft^37^. These studies may suggest inherent responses to such features, despite this evolutionary strategy being naturally limited to non-mammals, or responses may instead relate to species-specific social cues. Furthermore, woodcutters and other forest users have worn ornamental human face masks on the back of their heads in the Sundarbans in eastern India and western Bangladesh, with the intention of deterring ambush tiger (*Panthera tigris*) attacks^43^. The effectiveness of this approach remains undocumented in the scientific literature, however, and the potential for eyespots to deter predation of mammals more generally has not yet been assessed despite widespread potential application.

Livestock farming presents a clear need to manage predator–prey interactions. Globally, contemporary predator management still relies heavily on lethal control^44^, which in many cases has driven (e.g.^45,46^) or is driving carnivore populations to extinction^47^ and yet its effectiveness remains debatable (e.g.^48^). In regions where both livestock production and ecotourism are important for the economy, conflicts are almost inevitable, particularly where these industries are neighbours, such as at the edges of protected areas. While conflict prevention effort has often focused on improving livestock enclosures (e.g.^49,50^), livestock are usually only constrained within enclosures overnight if at all. Otherwise, livestock can roam freely while grazing, where the majority of predation may occur^51^.

In northern Botswana, on the fringes of the Okavango Delta World Heritage Site, non-commercial farmers operate small (mean ∼60 head) free-roaming livestock enterprises (“cattle-posts”) adjacent to protected areas (see Methods for study area description). The majority of reported predator attacks on livestock in the community (Shorobe) were by ambush predators—African lion (*Panthera leo*), and leopard (*P. pardus*)^51^, respectively—and farmers and herders direct a considerable and understandable degree of antipathy toward these predators^51^. Given the high tourism (e.g.^52^) and ecological value of these apex predators, there is a clear need to resolve these conflicts while also protecting large carnivores and rural traditions and livelihoods.

In this study, we tested whether conspicuous artificial eyespots applied to livestock would deter attacks by ambush predators. Our results suggest that artificial eyespots painted on to livestock were successful in deterring lions from attacking cattle. Our results also suggest that simple crosses were moderately effective, and together these results provide support for both the ‘detection hypothesis’ and the ‘conspicuousness hypothesis’ in this context.

## Results

### Effect of treatment on cattle survival

**Fig. 1:**
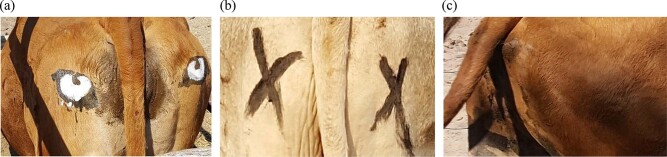
Experimental treatments applied to cattle. **a** artificial eyespots (bicolour as pictured, or white/yellow inner only, or black outer only, for maximum contrast depending on cattle coat colour). **b** cross-marked procedural control (black or white depending on coat colour for contrast). **c** unmarked control. Images provided by C.R.

To test whether attacks on free-ranging livestock by wild large predators could be prevented by painting artificial eyespots on cattle, we selected 14 cattle-posts (each with one cattle herd) that had reported high predation in recent months. Within each herd, adult cattle were assigned into one of three treatment groups: 1—artificial eyespots (Fig. 1a); 2—cross-marked (Fig. 1b); or 3—unmarked (Fig. 1c). During the study we undertook 49 painting sessions before the cattle were released from overnight fenced enclosures (interval between painting sessions mean = 29.61 days, sd = 14.33). We applied all three treatments during each painting session, and noted herd composition and predation events that had occurred since our last visit (see ‘Methods’ for description of painting and predation investigations) (Table 1).

**Table 1:** Total number of cattle within each treatment group (artificial eyespots, cross-marked, unmarked) in each year of the study and overall.

Year	Artificial eyespots (predations)	Cross-marked (predations)	Unmarked (predations)	Number of cattle
2015	70 (0)	0 (0)	125 (2)	195
2016	53 (0)	0 (0)	102 (5)	155
2017	305 (0)	293 (0)	344 (2)	942
2018	255 (0)	250 (4)	264 (6)	769
**Total**	**683 (0)**	**543 (4)**	**835 (15)**	**2061**

The number of predations by ambush predators (lion and leopard) are given in brackets.

*Predations by non-ambush predators (spotted hyaena: 1 predation in each treatment) were removed from the analysis*.

**Fig. 2:**
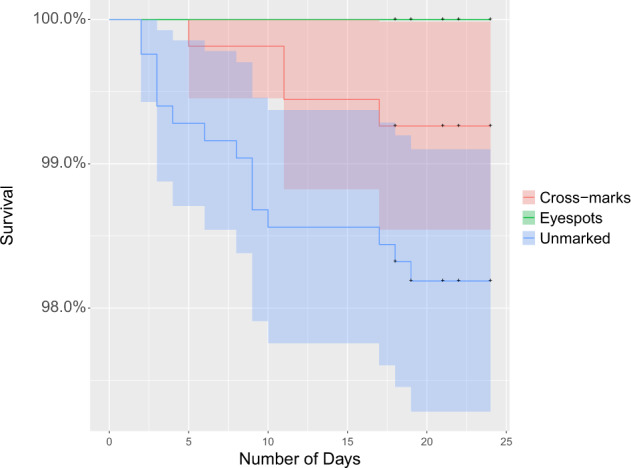
Ambush predators were less likely to kill cattle painted with artificial eyespots than unmarked (Df = 1, *p* = <0.001) and cross-marked (Df = 1, *p* = 0.011) cattle, and less likely to kill cross-marked than unmarked cattle (Df = 1, *p* = <0.001). Plot shows estimated percentage survival curve of cattle in each treatment (*n* = 683 cattle with artificial eyespots—green; *n* = 543 cross-marked cattle—red; and *n* = 835 unmarked cattle—blue), with 95% confidence bands from a survival analysis model. ‘+’ indicates time (days) when the treatment was reapplied. Treatment periods were for a maximum of 24 days, repeated cyclically.

Our results suggest that artificial eyespots were successful in deterring ambush predators (lions and leopards) from attacking cattle on which they were painted during the study period (Fig. 2). Ambush predators killed no artificial eyespot-marked cattle (*n* = 683, 0%) in the study herds, while four procedural control (cross-marked) (*n* = 543, 0.75%) and 15 unmarked (*n* = 835, 1.80%) cattle were killed by lions (*n* = 18) or leopards (*n* = 1) during the study (Table 1; Table 2). There was strong evidence to suggest an overall effect of treatment on survival of cattle (Df = 2, *p* = <0.0001) (Table 3). The subsequent pairwise comparisons of treatments indicated a strong difference between artificial eyespots and unmarked (Df = 1, *p* < 0.0001), and artificial eyespots and cross-marked (Df = 1, *p* < 0.011); however, there was also evidence for a strong difference in survival between cross-marked and unmarked (Df = 1, *p* < 0.001 ) (Fig. 2) (Table 4). As only 1/19 predation events involved a leopard, it is likely that this result is driven by lions.

**Table 2:** Predation events of cattle by large predators (lion, leopard and spotted hyena) in three treatment groups (artificial eyespots, cross-marked, unmarked).

	Treatment	Lion	Leopard	Spotted hyaena	Total
Number of predation events on cattle during the study period	Artificial eyespots	0	0	1	**1**
	Cross-marked	4	0	1	**5**
	Unmarked	14	1	1	**16**
	**Total**	**18**	**1**	**3**	**22**

**Table 3:** Overall effect of artificial eyespots on survival of cattle from ambush predators.

Predictor Variable	Likelihood Ratio Test Statistic	Df	*P* value
Overall treatment effect	35.0732	2	2.421e−08***

***p = <0.001; *N* = 139 (48 artificial eyespots, 43 cross-marked, 48 unmarked; number of times each treatment was applied to each herd of cattle at each cattle-post). Mixed effects cox regression investigating whether artificial eyespot treatment affects predation by ambush predators (lion and leopard). Cattle-post and cattleID were included as random terms to control for repeated measures, and treatment and herd size were included as covariates.

**Table 4:** Pairwise comparisons of artificial eyespots with other treatments for predation by ambush predators.

Pairwise comparison	Likelihood ratio test statistic	Df	*P* value
Artificial eyespots—cross-marked	6.466	1	0.0109956*
Artificial eyespots—unmarked	27.7774	1	4.00e−07***
Cross-marked—unmarked	14.68	1	1.911e−04***

^*^p = <0.05, ***p = <0.001. Comparison sample sizes: artificial eyespots versus cross-marked (*N* = 91; 48 artificial eyespots, 43 cross-marked); artificial eyespots versus unmarked (*N* = 96; 48 artificial eyespots, 48 unmarked), and cross-marked and unmarked (*N* = 91; 43 cross-marked, 48 unmarke). Cattle-post and cattleID were included as random terms to control for repeated measures, and treatment and herd size were included as covariates.

### Exposure to risk

**Fig. 3:**
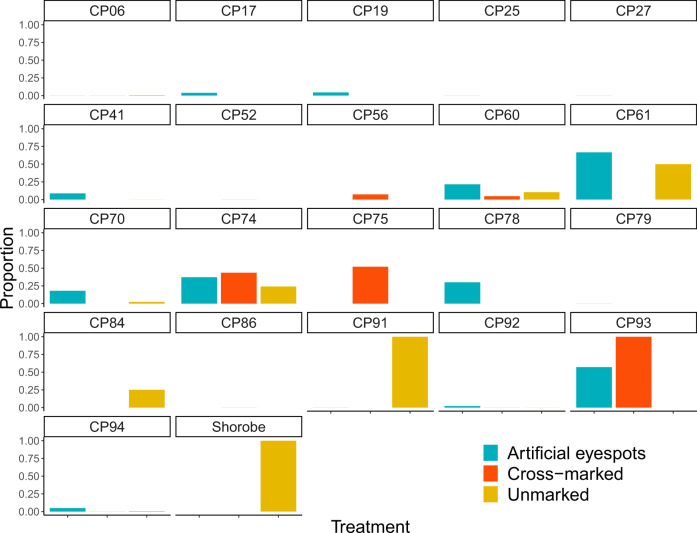
Cattle from each treatment group spent a similar proportion of nights outside their overnight enclosure overall. Bar graphs showing the proportion of nights that GPS-collared cattle spend outside each cattle-post (CP) during the study period (estimated from fixes collected between 00:00–01:00). Aqua bars represent artificial eyespot treatment group, orange bars represent cross-marked treatment group, and yellow bars represent unmarked treatment group. High proportions of nights outside for the unmarked treatment in two cattle-posts (CP91 and Shorobe) and the cross-marked treatment in another cattle-post (CP93) were due to these cattle not being herded or kraaled and, hence free-ranging across the landscape. A generalised linear mixed model with a binomial error structure was used with the proportion of cattle outside (00:00–01:00) as the response variable, and treatment (artificial eyespots, cross-marked or unmarked) as the predictor variable. Overall, there was no evidence to suggest there was a difference between treatment groups (DF = 2, *p* = 0.111).

**Fig. 4:**
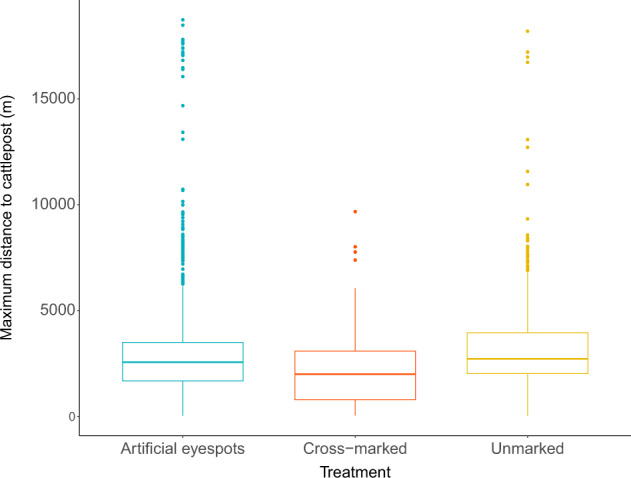
Cattle from each treatment group did not travel different maximum daily distances from their cattle-post (Df **=** 2, *p***=** 0.572). A linear mixed model provided no evidence for a difference between treatments in the maximum daily distance of the GPS-collared cattle from the overnight enclosure. Higher maximum daily distances (metres) were considered more exposed to predation risk. The box range represents the IQR (Q1 to Q3), the whiskers represent the Q1–1.5*IQR to Q3+1.5*IQR. The dots represent outliers outside this range.

There was no evidence to suggest these effects were due to differential exposure of each treatment group to predators. Cattle from each treatment group were fitted with GPS-logger-collars, and each treatment spent a similar proportion of nights outside the cattle-post (Df = 2, *p* = 0.111) (Fig. 3), and ranged to similar maximum daily distances from the cattle-post (Df = 2, *p* = 0.572) (Fig. 4) (see “Methods” for details of exposure of cattle to predation risk).

Collectively, these results suggest that these simple, low-cost treatments were effective in deterring ambush predators from attacking unattended free-ranging cattle.

## Discussion

The relative effectiveness of artificial mark types in this experiment allows us to speculate on the mechanism of their anti-predator effects, shedding new light on the evolution of anti-predator signals more generally. As predation of cattle with artificial eyespots was lower than cross-marked cattle, and predation of cross-marked cattle was lower than unmarked cattle, we found no support for the ‘deflection hypothesis’. This is because the deflection hypothesis states that attacks would be deflected to other body regions on cattle painted with eyespots (or cross-marks), which would not have reduced attacks on eyespot- or cross-mark-bearing cattle overall^2,12–15,17,18^. Given there was evidence for the cross-mark treatment being effective in deterring ambush predation as well, there remains the potential that paired eyespots and cross-marks may simply intimidate predators because they are novel or rarely encountered conspicuous features^24–27^. Nevertheless, the treatment providing the strongest evidence for increased survival of cattle was the artificial eyespots. This finding suggests that the anti-predation effect in this experiment is most strongly related to the eye pattern itself, or perhaps the aposematic coloration of white or yellow against black in a paired pattern.

Of the key hypotheses suggested to explain the evolution of eyespots in other taxa, our experiment provides some support for the ‘detection hypothesis’, the ‘predator-mimic hypothesis’, and the ‘conspicuousness hypothesis’. Distinguishing between these hypotheses is problematic, however, as predicted responses are similar, and the mechanistic difference is one of perception.

The ‘detection hypothesis’ predicts that eyespots signal to would-be attackers that they have been detected by their intended target, dissuading further attack (known as ‘pursuit deterrence’)^33–35^. It has been hypothesised that eyespots on the nape of some small raptors such as pygmy owl (*Glaucidium* spp.) and American kestrel (*Falco sparverius*) convey vigilance, dissuading ambush attacks by other raptors^32,33^. Hares stand erectly and directly facing approaching foxes to indicate to the fox that it has been detected^53^. Trinidadian guppies (*Poecilia reticulata*) are significantly less likely to be attacked by predators after ‘inspecting’ them^54^. More generally, many prey species stare directly at ambush predators when detected, to dissuade the predator from further attack because the element of surprise has been lost. This vigilant staring behaviour also allows other potential prey in the vicinity to determine where the predator is located, further dissuading the predator from attacking^34,35^. Both lion and leopard are ambush hunters that rely on remaining hidden from their prey. While solitary hunting leopards almost exclusively utilise an ambush hunting strategy, and require vegetation for concealment and to cache their prey^55^, lions are more flexible, either ambushing, or rushing their prey^56^, or employing a combination of these strategies depending on the habitat, social hunting group, and the target prey^56,57^. In this study, the predominant habitats were a mosaic of dense mophane woodland (*Colophospermum mopane*), mixed acacia shrubland (*Acacia* spp.) and riverine forest, and as all predation events were located within 10 m of shrubs or dense woodland it seems plausible that ambush was the likely method of attack, providing an appropriate context for the ‘detection hypothesis’ as a logical anti-predation mechanism. We reiterate here that there were too few predation events by leopards for an independent analysis, and so our results are predominantly driven by lions.

The ‘predator-mimic hypothesis’ suggests that eyespots have evolved to mimic the eyes of an enemy of the attacker, thus intimidating the would-be attacker^2,8,19–23^. Although the artificial eyespots in this study were too large, far set and anteriorly set to mimic any natural enemy of leopards and lions, it remains possible that the artificial eyespots represent a novel intimidating potential enemy not previously encountered. Such an effect has been shown in birds, where unnaturally large eye markings effectively deterred birds from baled silage while large crosses did not^58^.

Our experiment provided evidence for increased survival of cattle bearing the cross-mark treatment compared to unmarked cattle. While such an effect would not be expected under the ‘predator-mimic hypothesis’, it does provide some support for the ‘conspicuousness hypothesis’. Mukherjee and Kodandaramaiah^29^ showed that paired, fanned non-eye-like patterns were just as effective as eye-like circular patterns at deterring attacks from chickens. Critically, they found that models with a pair of patterns, regardless of symmetry or shape, received fewer attacks. As it has been shown that deterrent signals contrasting with the background cause rapid avoidance learning^59,60^, the cross-marks we applied in this study were either black or white to maximise contrast against the coat colour of the individual cattle. In the context of implementation as a predator deterrent in a predator-livestock conflict setting, the result that simple crosses may increase livestock survival is intriguing, as it suggests that markings may not need to be detailed eyespots, which may be challenging for everyone to recreate in situ.

In relation to the above hypotheses, it is also worth noting that in our experiment one individual from each treatment was killed by spotted hyaena, *Crocuta crocuta* (Table 1). While selective attacks by this and other non-ambush predators could shed light on the function of artificial markings on cattle survival, particularly regarding the ‘predator-mimic’ or ‘conspicuousness’ hypotheses, our limited data provide no such distinction, as we found no evidence of particular treatments being selectively preyed upon. Furthermore, as spotted hyaena are a cursorial, non-ambush predator, they offer no insights into the ‘detection hypothesis’, as detection by prey is unlikely to affect their predatory attempts. Certainly, a great deal of additional data are required from cursorial predator attacks to draw any firm conclusions on the mechanism of deterrence, if any, in this context.

Collectively, while we found no evidence to support either the ‘deflection hypothesis’ or the ‘predator-mimic hypothesis’, we could not fully discount the remaining two hypotheses. Although the measured effect of cross-marks was lower than the effect of eye-patterns in increasing cattle survival, which provides some support for the ‘detection hypothesis’, the increased survival of cross-marked cattle relative to unmarked cattle in the experiment also provides some support for the ‘conspicuousness hypothesis’. As these hypotheses are not mutually exclusive however, it is possible that multiple mechanisms are at play in this context.

As human settlements and livestock enterprises continue to border or even expand into areas important for large predator populations (e.g.^61^), human-wildlife conflict is an increasing occurrence^62^. Our results demonstrate that paired artificial eyespots and—to a lesser extent—paired cross-marks deter ambush predators from attacking cattle bearing these markings. Accordingly, we suggest that this inexpensive and easily implementable livestock-protection tool could be applied to high-value cattle within herds in any system affected by ambush predators. Particularly, they appear to protect cattle from attack in a landscape where cattle roam unprotected, and the additive value of this technique, in combination with herding and other husbandry, could be considered to facilitate greater coexistence between large predators, humans and their livestock. Whether whole herds painted with artificial eyespots or cross-marks deter ambush predators requires further testing; however, it is likely that a combination of both treatments (representing two concurrent anti-predator mechanisms) would be more effective than one or the other in isolation, at least in delaying habituation. Whether this technique is applicable to other predator species, over longer periods, or in wet or other hostile conditions, also remains to be tested. When used however, it is recommended that the technique be applied periodically, when predation rates are higher, also to avoid predator habituation. In combination with other techniques, successful implementation of this technique may increase the tolerance of farmers towards large predators, reduce the application of lethal control, and increase the sustainability of the system overall.

## Methods

### Study Area

The study was undertaken with the support of the Department of Wildlife and National Parks (DWNP) Problem Animal Control (PAC) unit within the Ministry of Environment Wildlife and Tourism. Fieldwork was conducted under the Botswana Predator Conservation Trust’s (BPCT) long-running large predator research programme in northern Botswana (Research Permit EWT 8/36/4 XXXVIII (14)), and approval was granted by UNSW’s Animal Care & Ethics Committee (approval number 17/51 A).

The ∼1300 km^2^ study area encompasses a rural livestock area abutting a protected area (the south-eastern region of the Okavango Delta UNESCO World Heritage Site), including 103 cattle-posts and their grazing land, with >2100 head of cattle^52^. These livestock farming areas surround the villages of Shorobe, Shukamukwa, Sexaxa, Morutsa and the Wildlife Management Areas bordering them: NG32, NG34, NG33, NG41 and NG47. The Okavango delta is one of the few remaining places on earth with an intact large predator guild, which persists, for now, alongside non-commercial livestock farming enterprises, but predator-livestock conflict is common in the region^51,63^.

The vegetation within these areas is a mix of mophane woodland and mixed shrubland, often bordering and including areas of fertile secondary and tertiary floodplains and dense riverine forest habitat. Africa’s large predators occur throughout these habitats, however ambush predators relying on vegetative cover and high prey densities often prefer denser vegetation with water associations and increased prey density for ambush opportunities^64,65^. When the natural prey of large predators disperse during the wet season (November–February), a shift in preference for livestock occurs (as reported by local farmers^51^). These predators are often killed in retaliatory lethal control following livestock predation events^51^. Between 2013 and 2015, the DWNP officially recorded 67 predation events in the Shorobe livestock farming area^51^. 82% of these events involved lions, and 13% leopards, with the remaining reports involving African wild dog (*Lycaon pictus*), cheetah (*Acinonyx jubatus*), spotted hyaena and black-backed jackals (*Canis mesomelas*)^51^.

### Cattle and painting

We selected 14 cattle-posts and herds (6–110 head of cattle in each) with recent high predation rates and owners willing to participate in the study. We consulted and informally engaged cattle-post owners on cattle and predator behaviour and activity around their cattle-posts.

During July–October 2015 and August–October 2016, August–December 2017, and April–November 2018, we painted paired artificial eyespots on the rumps of members of each herd after being herded into a cattle crush during the first few hours post-sunrise before cattle were released for the day. We applied acrylic paint (black and white or yellow) to foam stencils in the shapes of the inner and outer eye respectively, which had been glued to a wooden plasterer’s float. These colours were chosen because of their highly contrasting and aposematic features, common in natural anti-predator signalling settings^66^. On cattle with very dark coats, only the white/yellow inner eye stamps were applied, while on white cattle only the dark outer eye pattern was usually applied. Eyespots were applied to each side of the cattle’s rump whilst it was stationary within the crush (one eye on each side, applied by an observer reaching through the crush; Fig. 1a, Supplementary Information, Supplementary Movie 1, Supplementary Movie 2). A procedural control for the effect of paint and processing (a painted cross-mark) was introduced during the 2017 field season and continued through 2018. The delayed introduction of this treatment did not compromise or bias our results, as it was accounted for in the survival analysis (see ‘Methods’—‘Statistical Analysis’). Black or white crosses were painted depending on which provided the best contrast with the cattle coat colour, and were of a similar size, colour and position to the artificial eyespots. Combined black and white crosses may have represented a better procedural control for some cattle, and should be considered in future applications.

For the majority of the study (from 2017), we painted approximately one-third of each herd with the artificial eyespots and one third with the control cross-marks. The rest of the herd (approximately one-third) was handled in the crush but left unmarked. Treatments for individual cattle were haphazardly selected during the random procession that cattle entered the crush to exit the overnight enclosure. We recorded identification features such as existing tag ID, coat colour, sex, age and distinguishing features such as horns for individual cattle whilst in the crush following painting of treatments and before being released. Finally, we recorded the number of cattle within each treatment and the entire herd. Animals used in the study were 609 male, 1024 female and 428 unrecorded sex adult or subadult cattle of predominantly Tswana breed (*Bos taurus africanus*). Throughout the study, a total of 683 were painted with eyespots, 543 were cross painted with crosses, and 835 were in the unpainted control group (Table 1). Despite variable wear, paint would typically remain clear and obvious until approximately 24 days post-application. Therefore, we replaced the paint approximately every four weeks during the study period between July 2015 and November 2018 (interval between painting visits mean = 29.61 days, sd = 14.33). We also recorded the date of each treatment application on individual cattle, and temporarily excluded herds from the study 24 days after painting if they had not had paint reapplied within this time period. If a herd was not re-painted within this time frame, then we (haphazardly) re-painted the herd on the next visit when they were included again in the study. No predation events occurred on cattle that were temporarily excluded from the study. Study herds were not processed during the rainy season months of December to February due to logistical constraints of researchers accessing the cattle-posts. Due to their being gaps in between yearly study sessions (nine months between the 2015 and 2016 study sessions; nine months between the 2016 and 2017 study sessions; and three months between the 2017 and 2018 study sessions), it is likely that the novelty of treatments did not wear off for locally occurring large predators throughout the study period.

We recorded survival of individual cattle in each treatment (artificial eyespots, cross-marked, unmarked) during frequent visits to the cattle-post of each study herd or following reports from cattle-post workers. Herders recognised most individuals within their herds and so were quickly aware when individuals were missing. We then investigated predation events from study herds to identify the individual cattle (and hence the treatment applied to determine if markings had the potential to be effective as a deterrent during the predation event). Herders aided us when tracking, and we recorded the date of the predation event and number of days since the most recent treatment had been applied. With the herders’ assistance, we also collected evidence to aid in the identification of predatory species. This included the bite location and distance between canine intrusions, as well as supplementary evidence such as spoor around the kill. Ambush predators occurring in the study area such as lion and leopard typically kill prey with a suffocating bite to the throat^56^. Furthermore, lions may attempt to bury the stomach contents of the prey to hide the scent from scavengers, and leopards often pull their prey into a tree, out of reach of predators^67^. Scavenger species such as spotted hyaena and black-backed jackal may have visited the carcass after the initial kill, however if typical feline canine intrusions around the neck of the cattle were present then it was assumed that this species made the initial kill as these intrusions are only made to kill the animal^56^. We only included predation events caused by ambush predators (lion and leopard) in the results assessing the effectiveness of the tool in deterring predation, but we also recorded predation involving non-ambush cursorial predators; in this case only spotted hyaena (Table 1). Other predators previously reported to kill livestock in the region include cheetah (ambush), African wild dog (cursorial), caracal (ambush; *Caracal caracal*) and black-backed jackal (cursorial), however none of these species were found or reported to have preyed upon livestock during the study period.

### Exposure of cattle to predation risk

To determine whether cattle in each treatment group were exposed to similar predation risk, we fitted individuals from each treatment group with a GPS-logger (CatLog Gen2—Catnip Technologies Limited) mounted on a custom-built collar to record movement data. GPS coordinates were set to record every 10 minutes between 06:00 and 19:00 LMT (when cattle were out of the cattle-post) and every three hours at other times (when cattle were usually resting within the cattle-post enclosure). Collars were left on cattle for up to 5 months after which they were removed, downloaded, recharged, and replaced on the herd.

Two levels of predation exposure were measured during treatment periods: proportion of nights spent outside the cattle-post and maximum daily distance travelled from the cattle-post. Number of nights spent outside the cattle-post was an indication of how often cattle were exposed to predation at night when predators were more active, and cattle were not protected by the cattle-post. Maximum daily distance from the cattle-post was an indication of how far cattle travelled from the safety of the cattle-post, and hence whether they were more exposed to potential predation.

### Statistics and reproducibility

All statistical analyses were performed in the program R (Version 3.5.2) and RStudio (Version 1.1.463, downloaded 15/01/2018). To measure the success of artificial eyespots (and/or cross-marks) as an ambush predator deterrent, we utilised mixed effects cox regression models using the *coxme* package^68^ to model predation hazard for different treatments. We did this by treating each re-application of treatment as a new ‘time to event’, which ended when either (i) a predation event occurred in the herd, (ii) treatment was reapplied or (iii) 24 days after treatment, which is when the treatment had begun to wear off to the extent that it was likely ineffective. It is not possible to conduct Kaplan–Meier survival curve estimation for mixed effects cox regression models, and so we plotted survival curves using the *survival*^69^ and *ggfortify*^70,71^ packages. We note that this may make confidence intervals appear under-estimated relative to what would be estimated for a mixed effects approach. Throughout the analysis, we accounted for repeated measures on the same individual cattle and herd with random intercepts. We also applied the number of cattle within a herd and the number of cattle within a treatment as covariates in the analysis. To test for an overall effect of treatment on predation risk, we used a likelihood ratio test. To subsequently test for pairwise differences among the treatment groups, we created treatment data subsets to allow for pairwise comparisons of treatments using likelihood ratio tests on the pre-established mixed effects cox regression models. Subset data pairwise comparisons we included were artificial eyespots - cross-marked, artificial eyespots - unmarked, and cross-marked - unmarked. This method was used as opposed to traditional pairwise Tukey tests, to avoid misleading tests and inflated variances from a lack of predation within the artificial eyespot group (similar tactics are applied for perfect separation in logistic regression e.g.^72^). Finally, we adjusted the *p*-values using the Benjamini & Hochberg method^73^.

To test if each treatment group had similar exposure to predation risk, movement data from cattle in all cattle-posts and treatment groups were compared. All fixes were standardised to 10-min intervals. Distance of each fix from the cattle-post of origin was formulated using Pythagoras theorem in R.

### Number of nights spent outside the cattle-post

Each night, cattle were kept within predator-proof enclosures for safety, and were released the following morning. To determine whether the individual cattle were contained in the enclosure overnight, we extracted the GPS fix occurring between 00:00-01:00 using the *lubridate* package^74^. *dplyr*^75^ was used to subset this data by location (in or out of overnight enclosure). Cattle that were moved between cattle-posts were designated to the previous cattle-post until it arrived at the next cattle-post for the first time. To account for GPS fix location error and to eliminate false positive detections, all extracted fixes within 200 metres of the cattle-post of origin were classified as enclosed, and all fixes farther than 200 metres were determined to be outside the enclosure area and more vulnerable to predation. Indeed, all fixes within 200 metres of the cattle-post were likely to be somewhat protected by herders and residents who resided close (<100 m) to the overnight enclosure. To determine if there was a difference between treatment groups in the number of nights spent outside the enclosure, we used a generalised linear mixed model with a binomial error structure where the proportion of cattle outside (00:00-01:00) was the response variable and treatment (artificial eyespots, cross-marked or unmarked) was the predictor factor variable, using the *glmmTMB* package^76^. Cattle-post ID and individual cattle ID were both included as random effects.

### Maximum daily distance from the cattle-post

We compared maximum daily distances from the cattle-post for each treatment using a linear mixed model where the maximum daily distance from the centre of the overnight enclosure was the response variable, and treatment (artificial eyespots, cross-marked or unmarked) was the predictor. Cattle-post ID and individual cattle ID were both included as random terms to account for repeated measures. We then used a likelihood ratio test to test for an overall effect of treatment on risk of exposure to predation. We subsetted the maximum daily distances from the cattle-posts using the *dplyr* package^75^, and ran linear mixed effect models using the *lme4* package with Gaussian error structure^77^.

All plots were created using the *ggpubr*^78^ and *ggplot2*^79^ packages.

### Reporting summary

Further information on research design is available in the Nature Research Reporting Summary linked to this article.

## Supplementary information

Descriptions of Additional Supplementary Files

Supplementary Information

Supplementary Movie 1

Supplementary Movie 2

Reporting Summary

## Data Availability

The datasets generated during and/or analysed during the current study are available in the Zenodo repository, [10.5281/zenodo.3877999]^80^. All figures have associated raw data. There are no restrictions imposed on data availability.

## Code availability

R code used in this study is available in the Zenodo repository, [10.5281/zenodo.3877999]^80^.
