# Supplementary material for: Artificial eyespots on cattle reduce predation by large carnivores
Source: Commun Biol. 2020 Aug 7;3:430. doi: 10.1038/s42003-020-01156-0 (PMC7414152; doi:10.1038/s42003-020-01156-0)
Supplement: Supplementary file 1 — Descriptions of Additional Supplementary Files [file 42003_2020_1156_MOESM1_ESM.pdf]

Descriptions of Additional Supplementary Files

Supplementary Movie 1: A video demonstration guide to the practical application of the "Eye-cow" technique for livestock protection (English Language)

Supplementary Movie 2: A video demonstration guide to the practical application of the "Eye-cow" technique for livestock protection (Setswana Language)
