# Supplementary material for: Artificial eyespots on cattle reduce predation by large carnivores
Source: Commun Biol. 2020 Aug 7;3:430. doi: 10.1038/s42003-020-01156-0 (PMC7414152; doi:10.1038/s42003-020-01156-0)

Botswana Predator Conservation Trust

Go dirisa “eye-cow” mo go sireletseng leruo

Using “eye-cow” for livestock protection

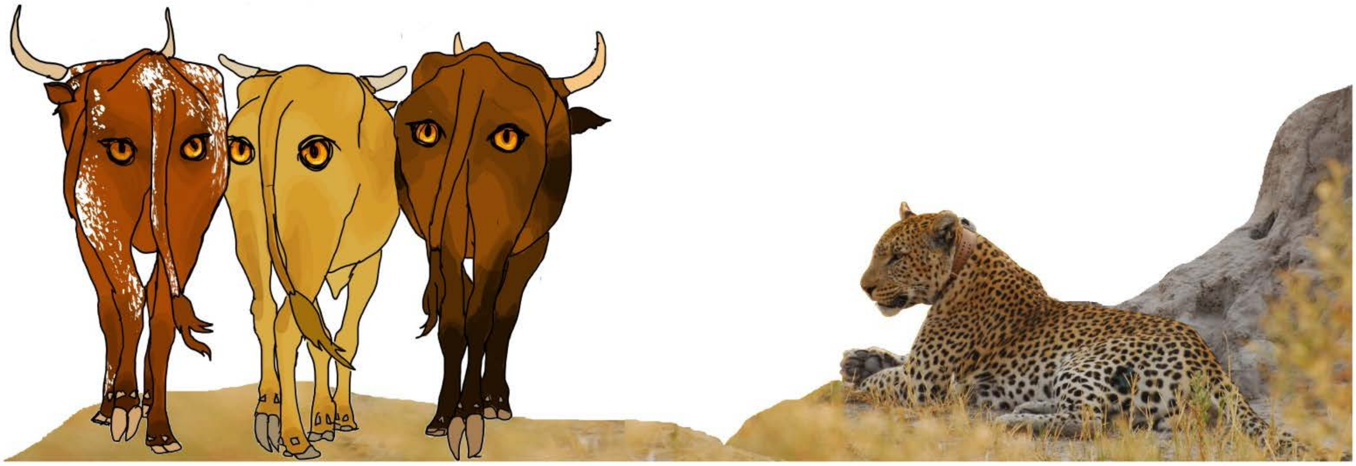

Artwork: Sam Lostrom

UNSW SYDNEY

TARONGA CONSERVATION SOCIETY AUSTRALIA For the Wild

Bobby-Jo Photography

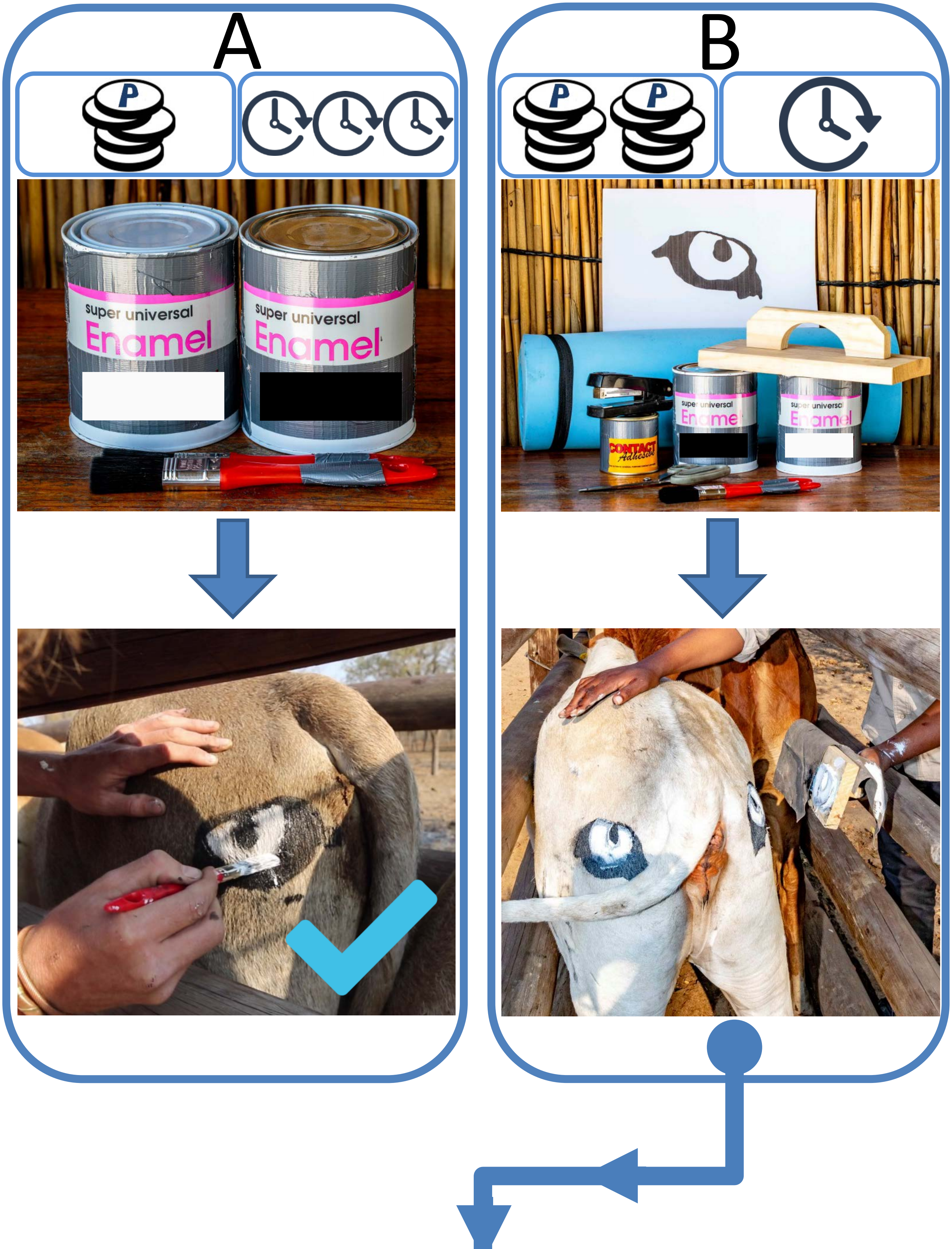
A
B
super universal
Enamel
CONTACT
Adhesive

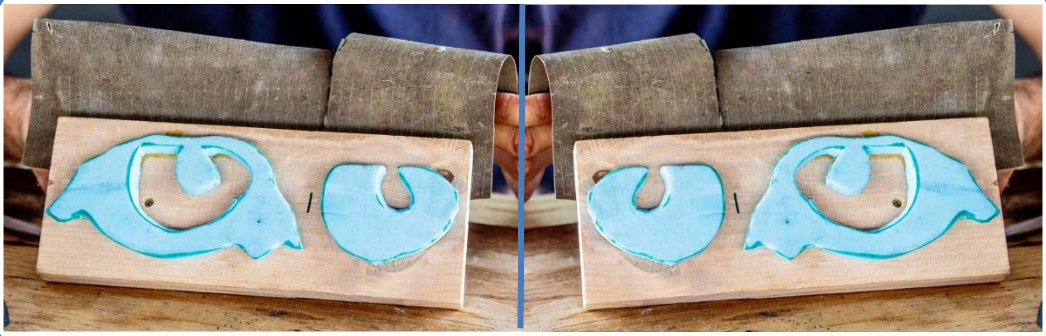

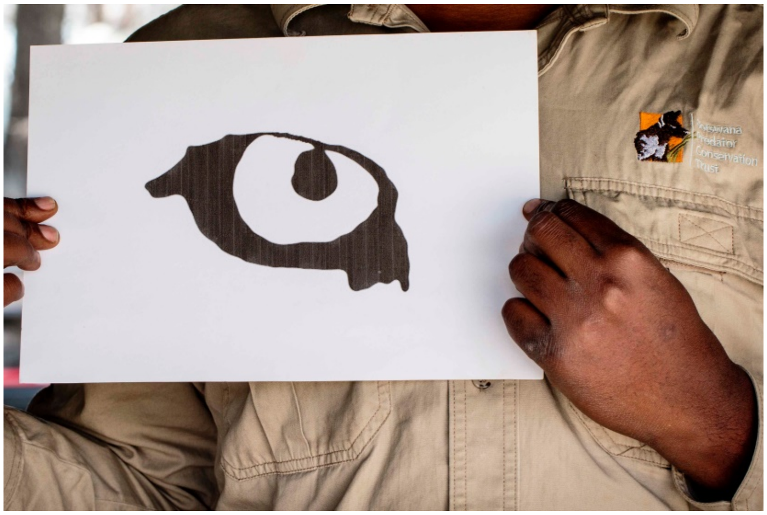

1

Otla bona setshwantsho sa leitlho ko bofelong jwa tshupegetso e.

Find the eye template at the end of this guide.

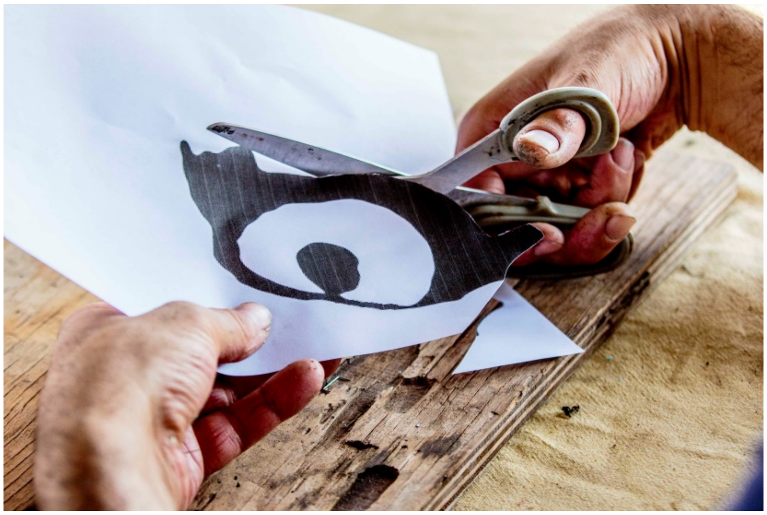

2

Segolola setshwantsho se.

Cut out template.

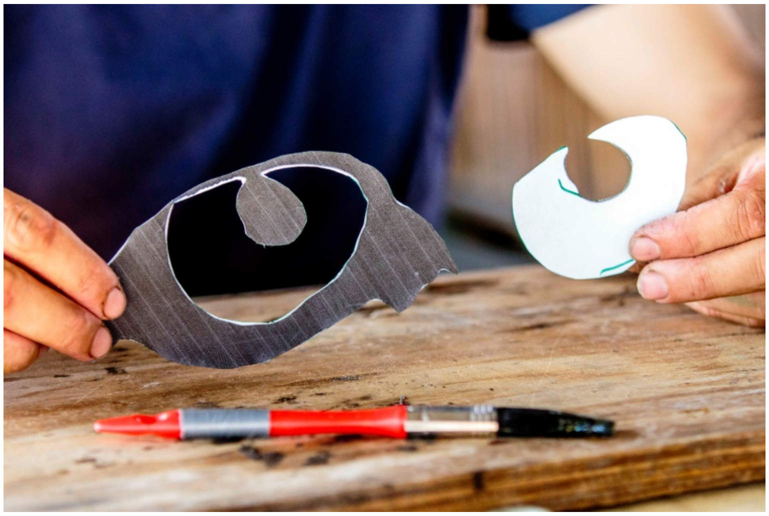

3

Fa o segolola setshwantsho oseka wa segolola fa gare ga sone.

Cut out and keep template centre.

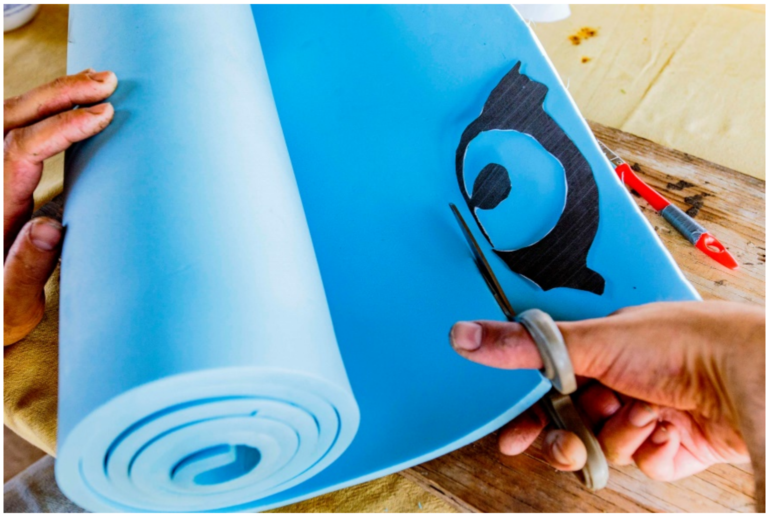

4

Sega leterase le le lekanetseng.

Cut correct size foam.

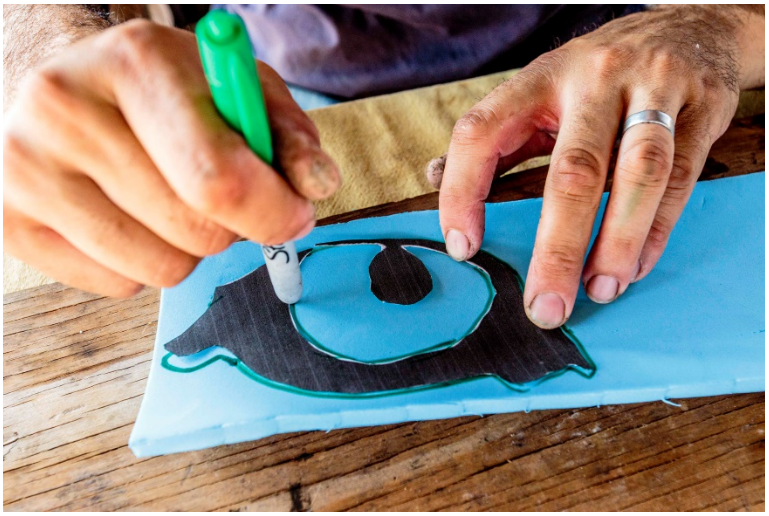

5

Tshwantsha go dikologa setshwantsho sa leitlho.

Draw around template.

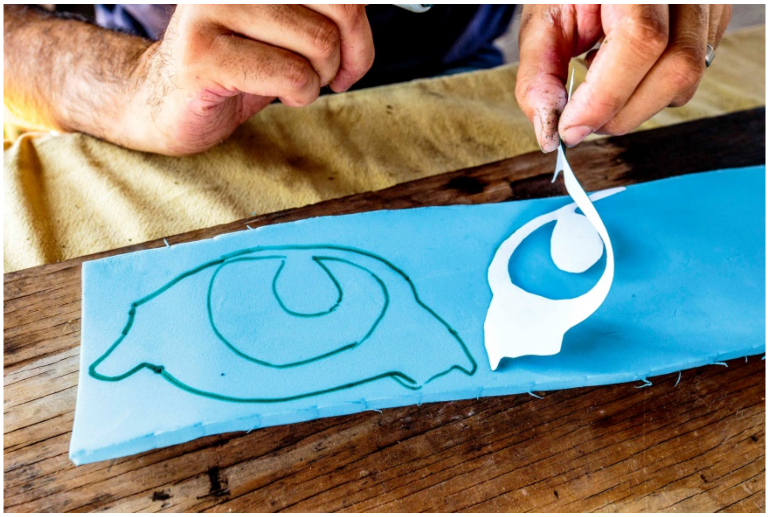

6

Pitikolola setshwantsho.

Flip template.

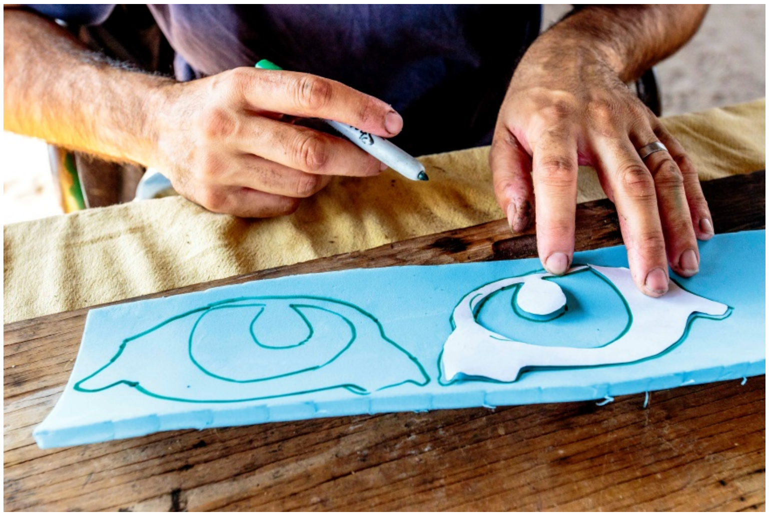

7

Tshwantsha o dikologa setshwantsho se se pitikolotsweng.

Draw around flipped template.

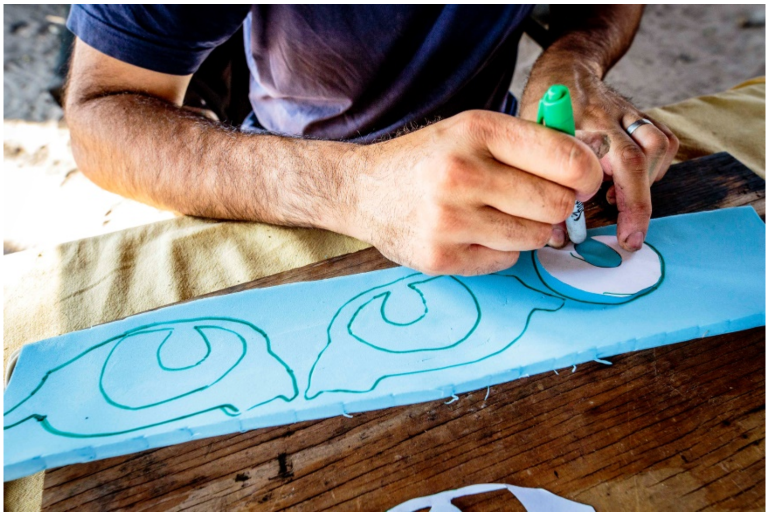

8

Tshwantsha o dikologa setshwantsho se se fa gare.

Draw around inner template.

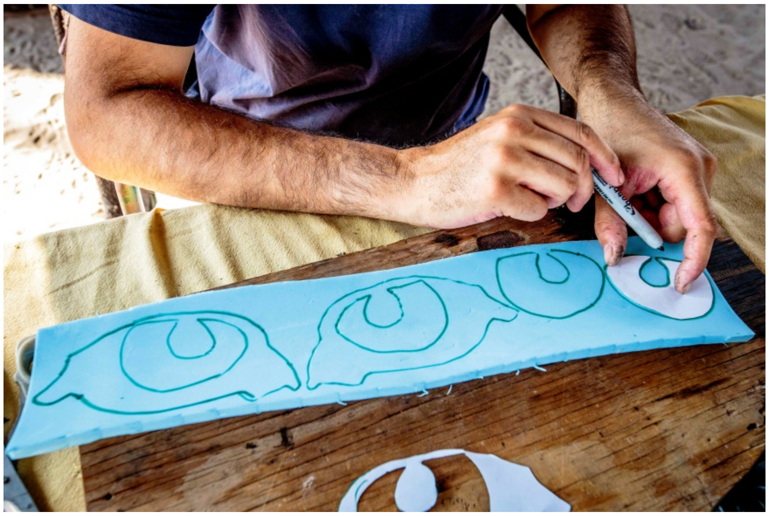

9

Pitikolola o bo o tshwantsha go dikologa setshwantsho se se fa gare.

Flip and draw around inner template.

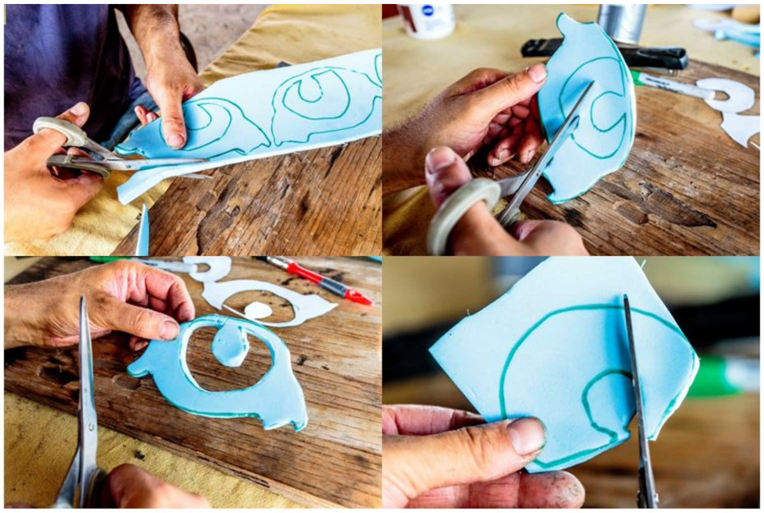

10

Segolola ditshwantsho tse tsotlhe ka bone jwa tsone.

Cut out all 4 patterns.

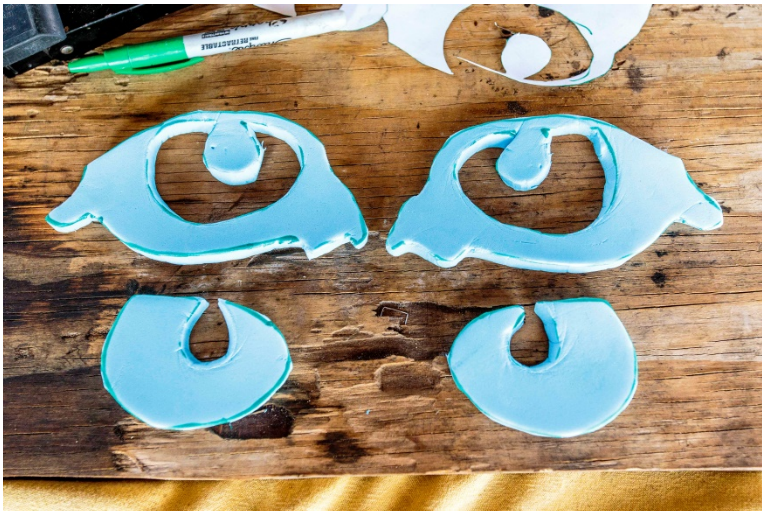

11

Di rulaganye ka go tsamaelana ga tsone.

Arrange in matching pairs.

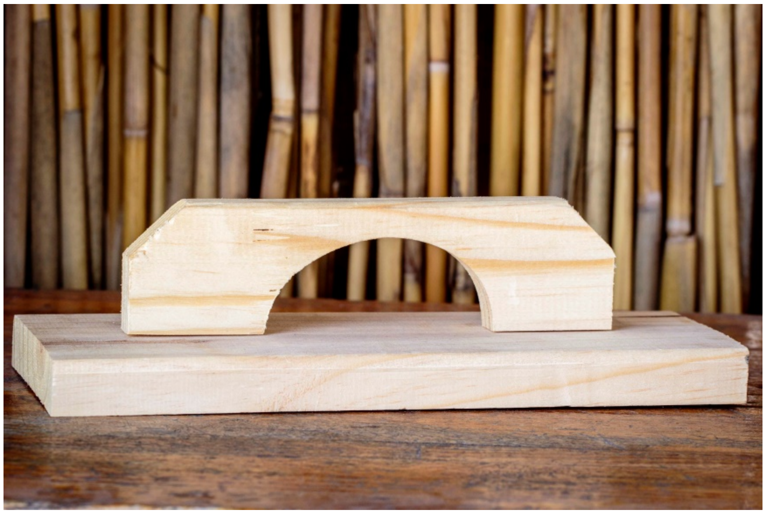

12 x2

Tsaya mapolanka ale mabedi a dirisiwang fa go dilwa ntlo.

Take 2 plasterers floats (or wood boards).

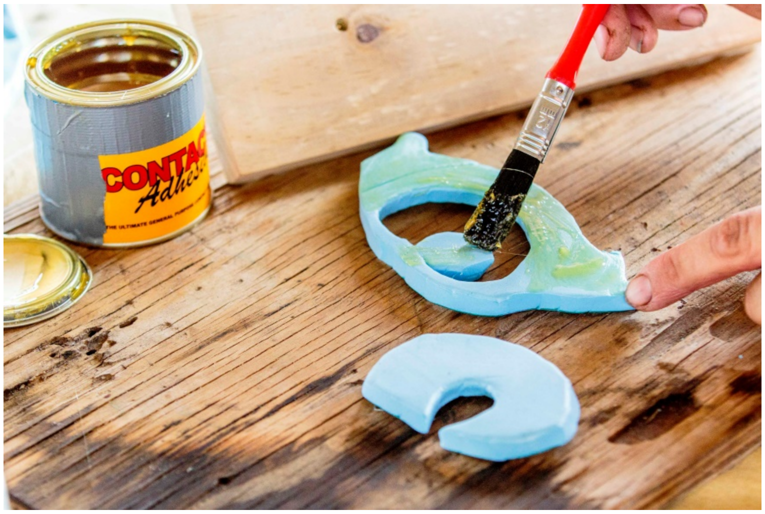

13

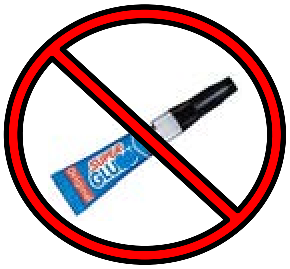

Tshasa leterase le ole segolotseng ko morago ka bongapa jo bo dirisiwang go ngaparisa ditlhako.

Paint the back of each foam cut-out with contact adhesive.

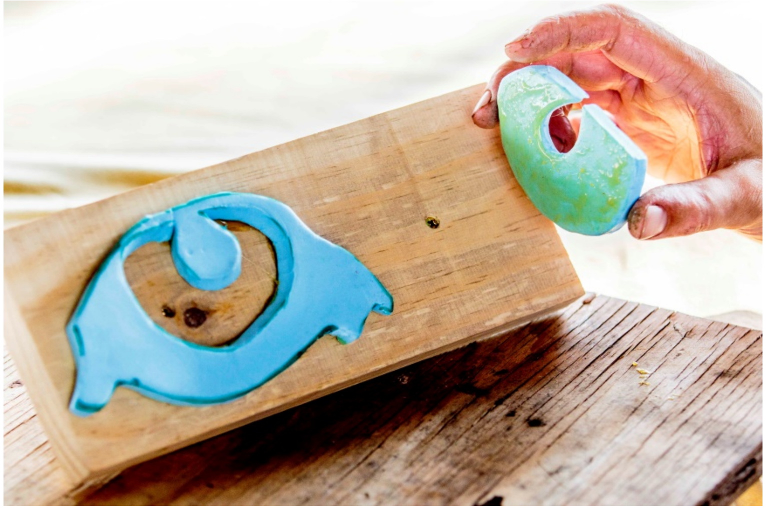

14

Ngaparisa ditshwantsho tse di tsamaelang mo lepolankeng lengwe le lengwe.

Stick a matching outer and inner pattern on each board.

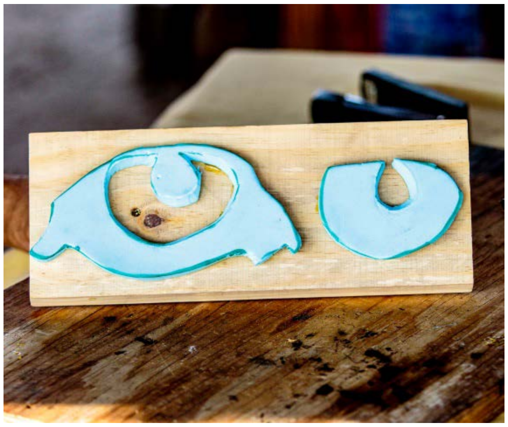
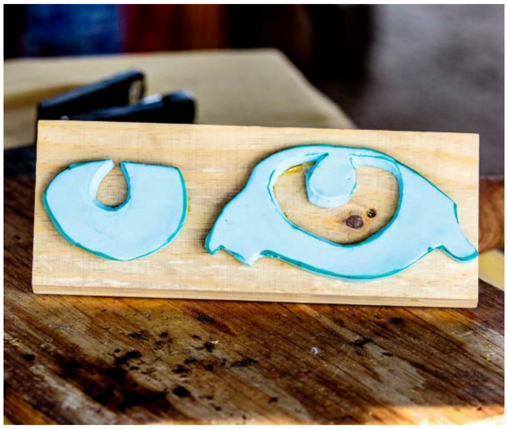

15

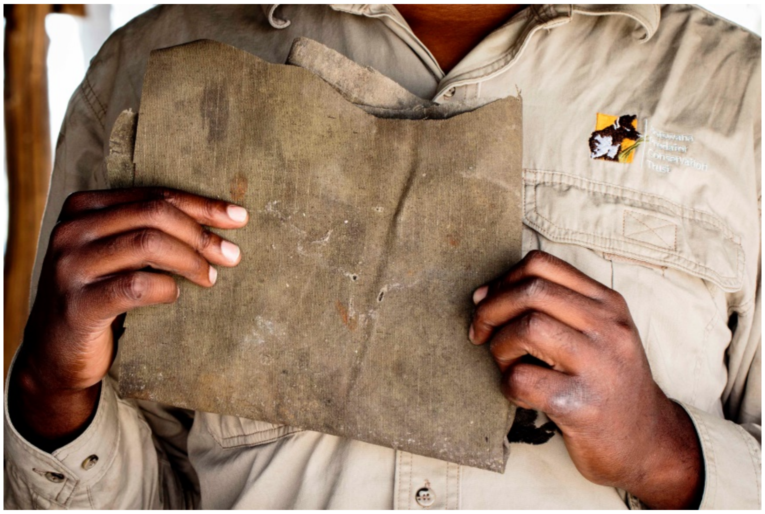

16

Batla letsela le le thata go ka khupetsa lepolanka ka lone.

Find old canvas (or similar) to cover board.

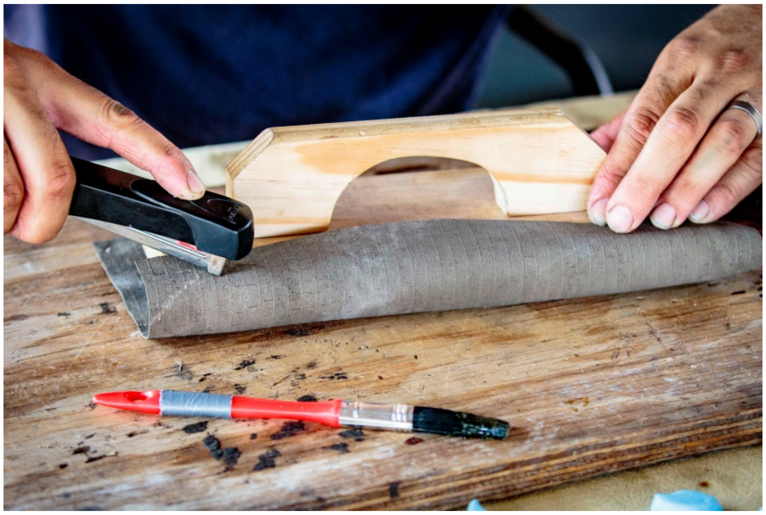

17

Ngaparisa letsela ko godimo, ele ko morago ga lepolanka.

Staple canvas edge to back of each board on 1 long side.

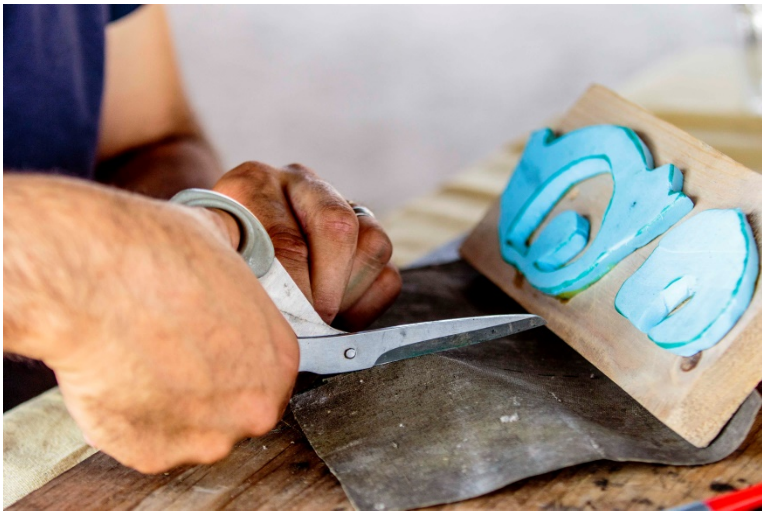

18

Gagola letsela fa gare ga ditshwantsho tsa leitlho.

Cut canvas between inner and outer eye patterns.

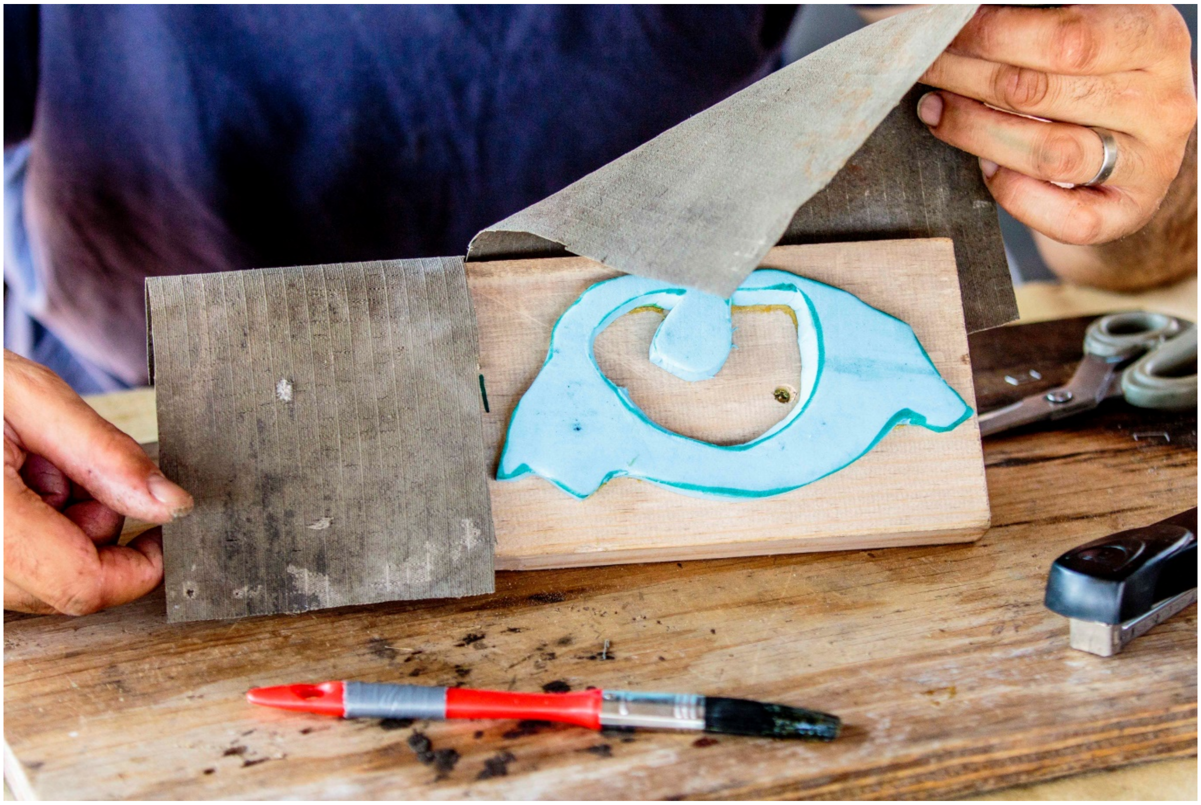

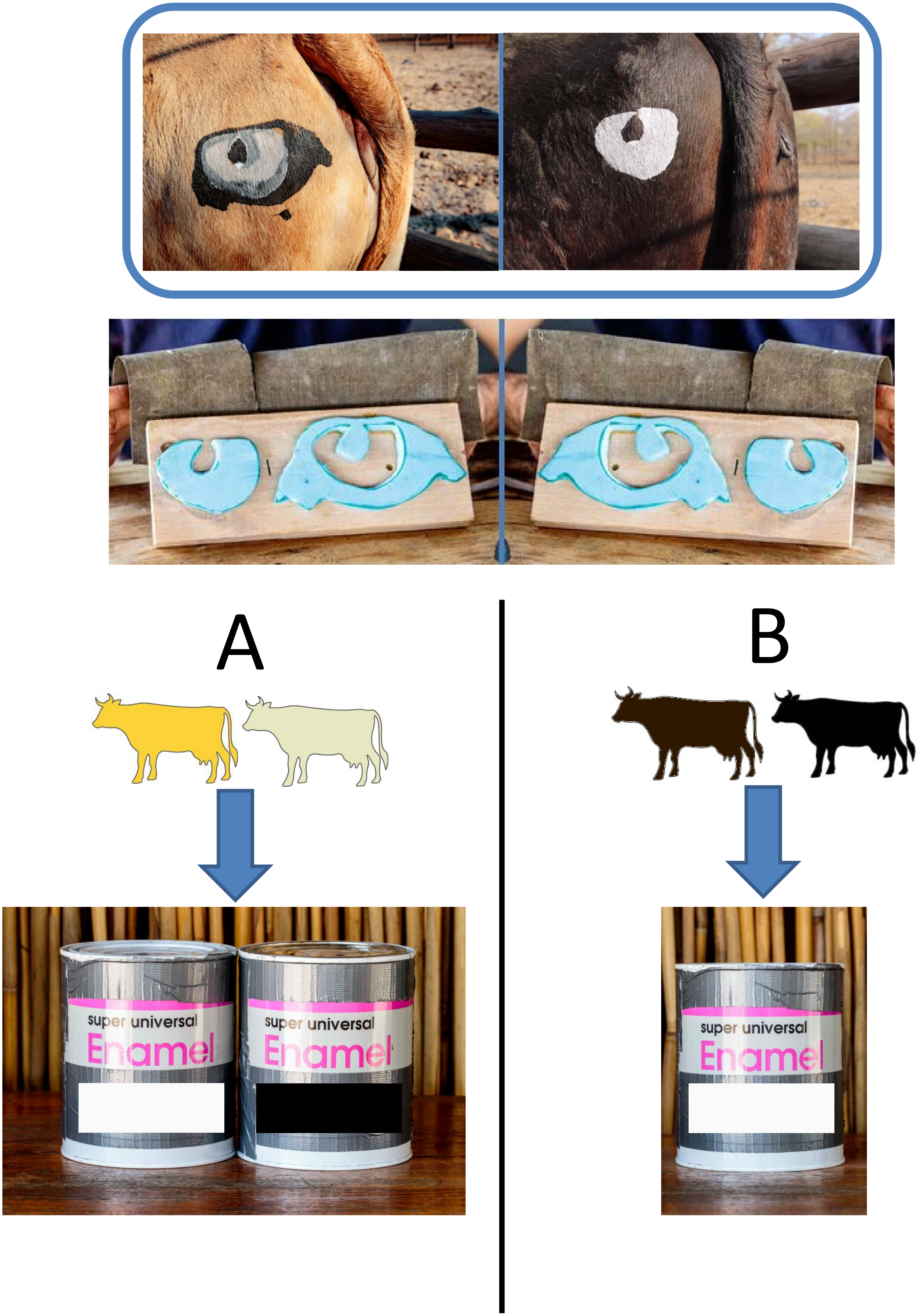
A
B
super universal
Enamel
super universal
Enamel
super universal
Enamel

A

1

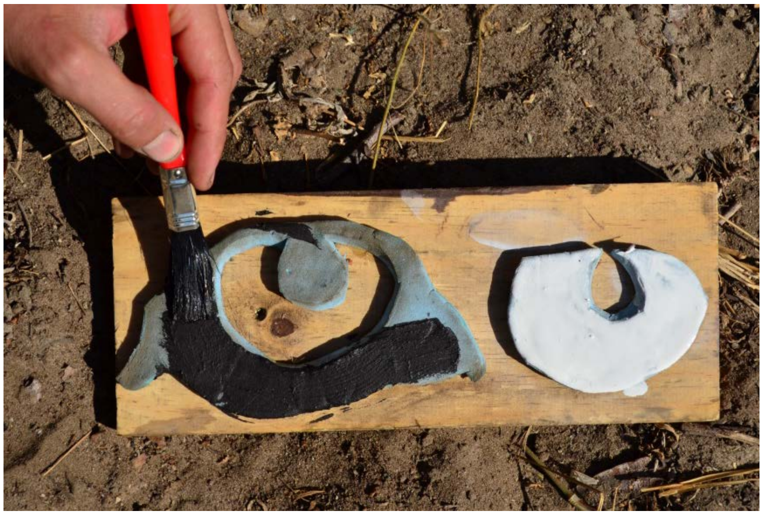

Mo leruong le le mmala mosetlha thata,dirisa mmala o montsho mo setshwantshong sa leitlho sa ko ntle le mmala o mosweu mo setshwantshong sa leitlho sa mo teng.

For pale livestock, apply black paint to outer eye, and white paint to inner eye patterns

2

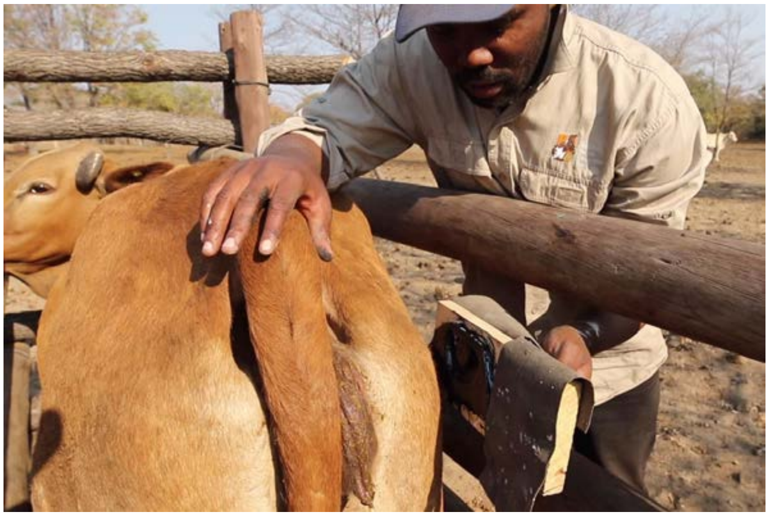

Pitikolola letsela go fitlha setshwantsho sa leitlho sa mo teng. Gatelela setshwantsho sa leitlho sa ko ntle,se se sa fitlhiwang ke letsela ko morago ga kgomo gangwe fela mo letlhakoreng lengwe le lengwe.

Flip canvas to cover inner eye pattern. Press and hold outer pattern on cattle rump; once each side.

B

1

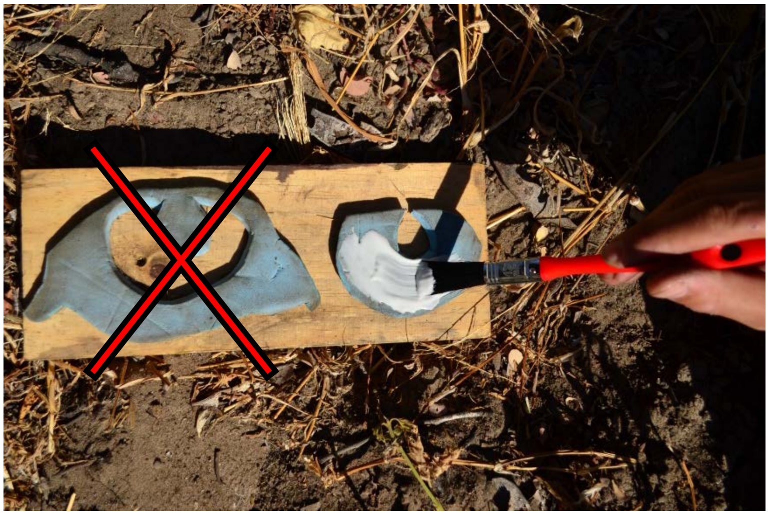

Mo leruong la mmala montsho kana bothokwa,dirisa mmala o mosweu mo setshwantshong sa leitlho sa fa gare.

For dark livestock, apply white paint to inner eye pattern only.

2

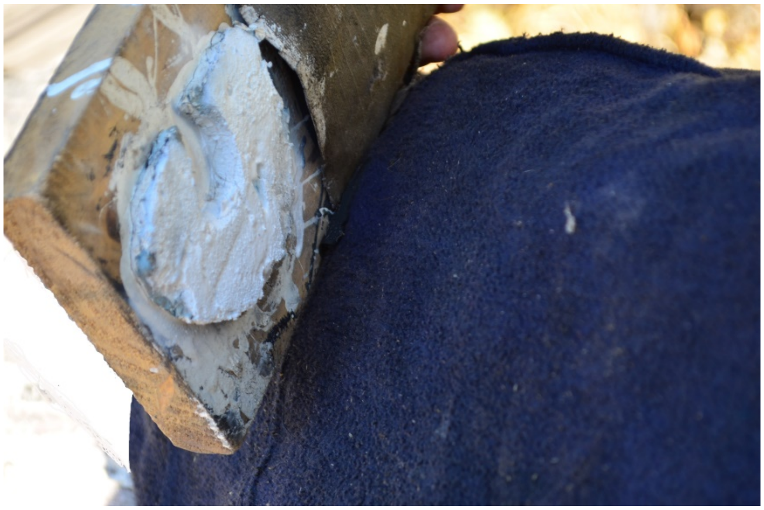

Gatelela setshwantsho sa leitlho ko morago ga kgomo gangwe fela mo letlhakoreng lengwe le lengwe.

Press and hold inner pattern on cattle rump; once each side.

A

B

3

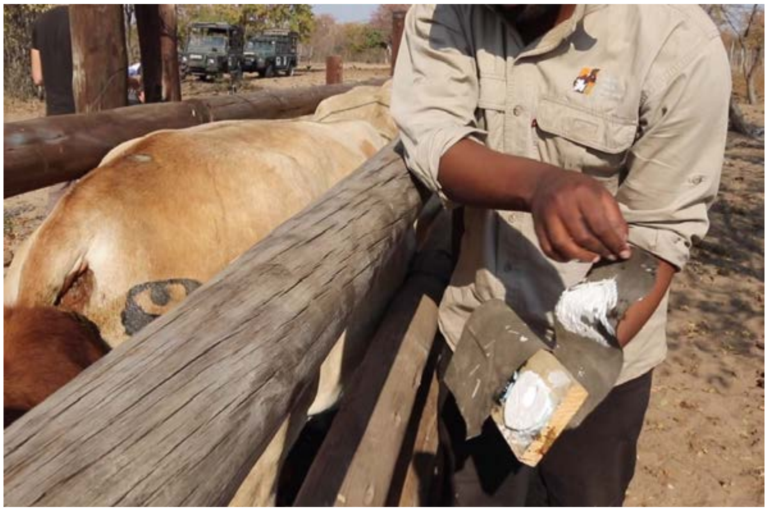

Pitikolola letsela go fitlha setshwantsho sa leitlho sa ko ntle, mme o fitlholole setshwantsho sa mo teng. Gatelela setshwantsho sa leitlho sa mo teng ko morago ga kgomo mo letlhakoreng lengwe le lengwe.

Flip canvas to cover outer eye pattern. Press and hold inner pattern on cattle rump.

4

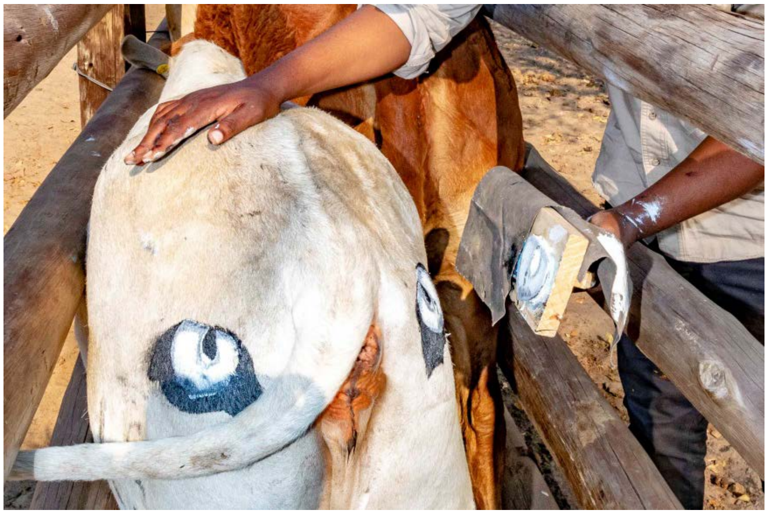

Dira gape mo letlhakoreng le lengwe.

Repeat on other side.

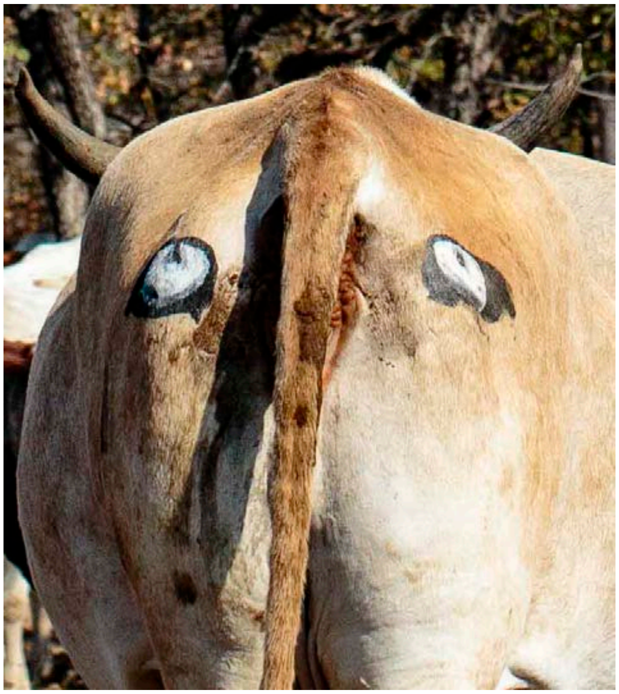

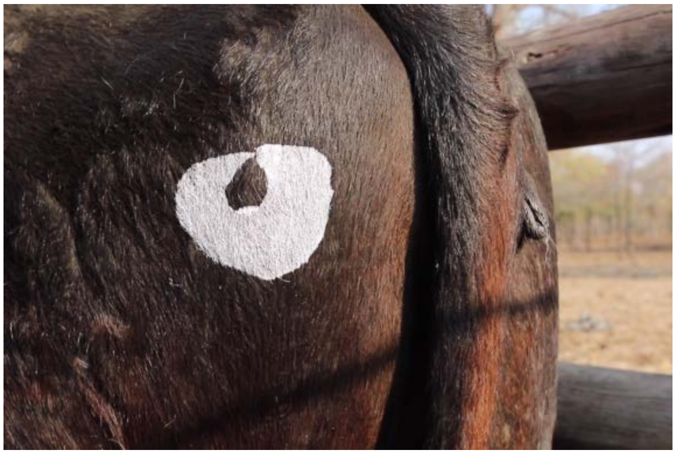

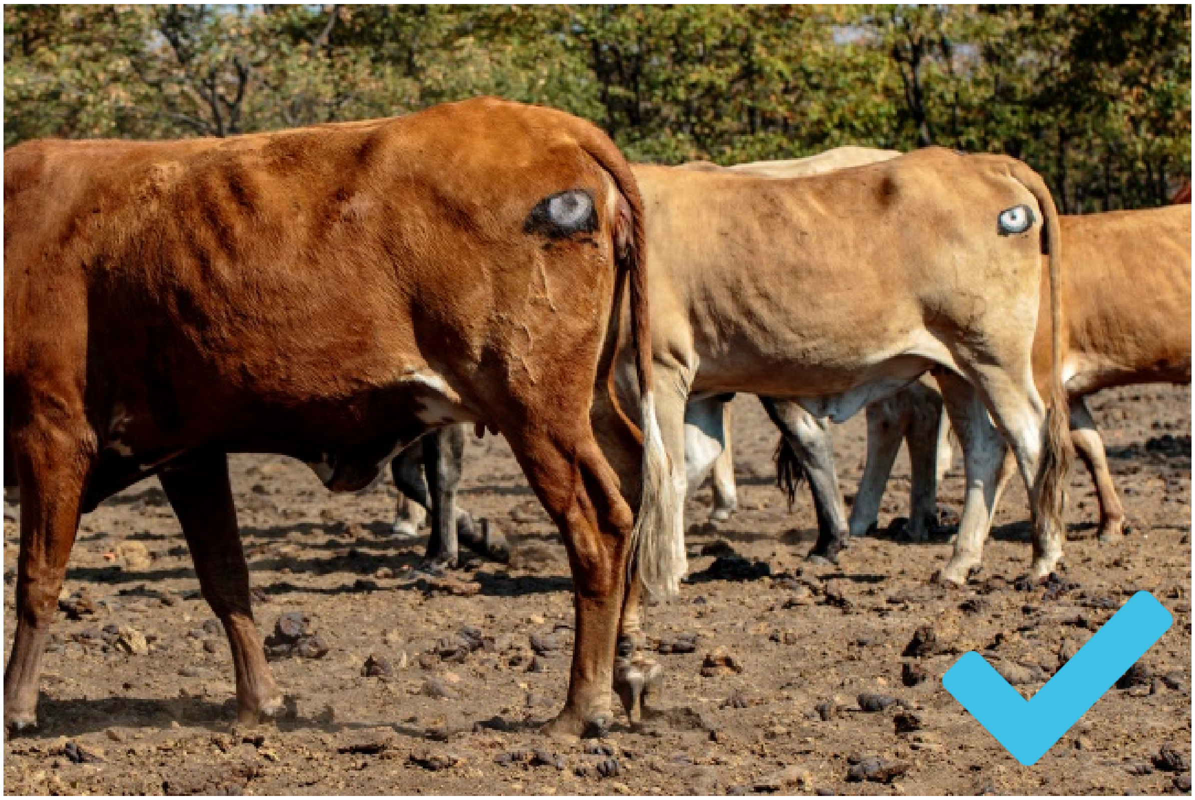

Ditshwantsho tse di tshasitsweng ko morago ga kgomo ditla sutlega mo tsamaong ya nako, mme oka tshasa gape (gongwe morago ga beke tse tharo).

Painted eyes will fade over time. Re-paint when faded (approximately every 3 weeks).

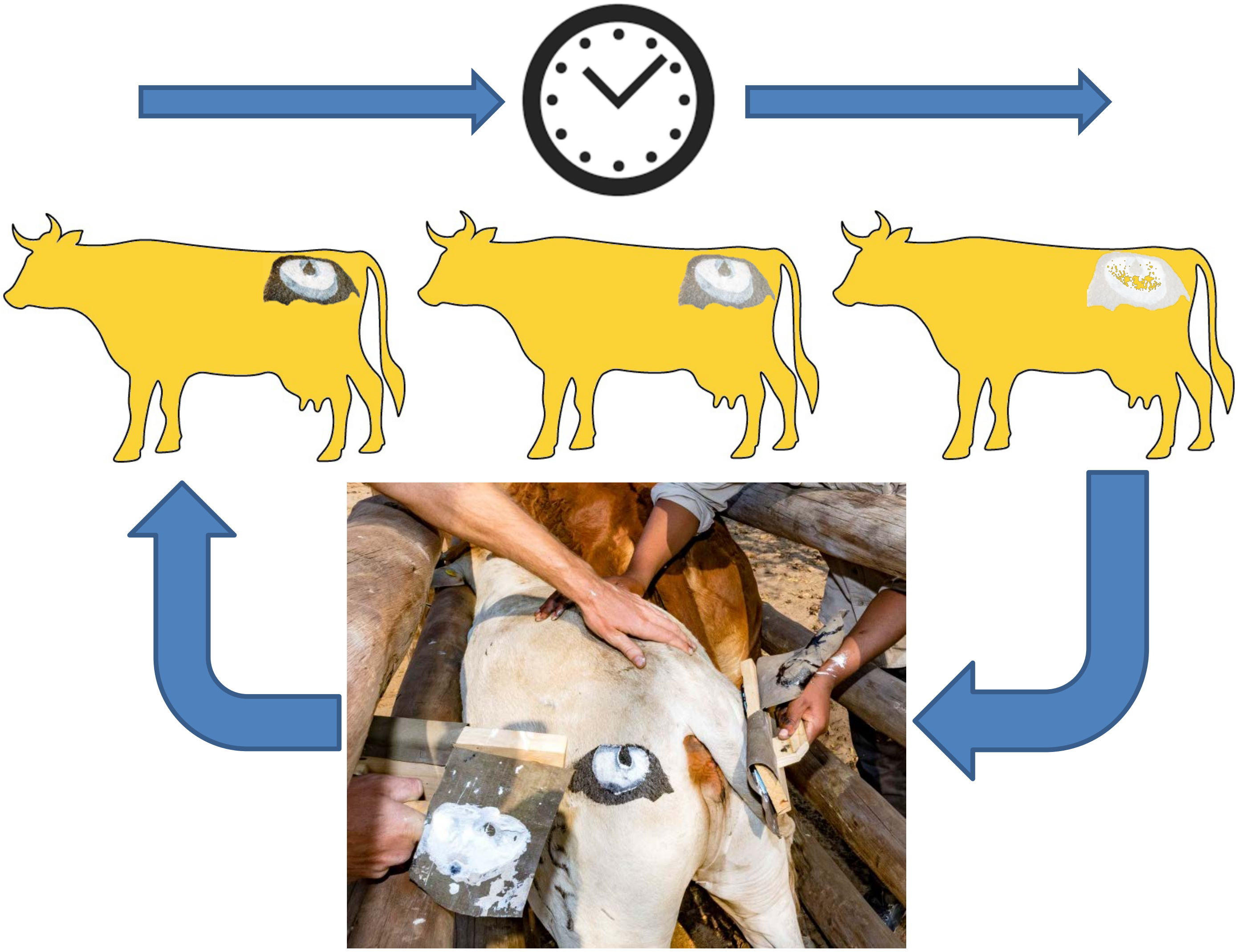

Botswana Predator Conservation Trust

experiment

UNSW SYDNEY

TARONGA CONSERVATION SOCIETY AUSTRALIA

For the Wild

Bobby-Jo Photography

Tshupegetso e,e dirilwe ke: **Ed van Mourik & Neil Jordan**

Guide produced by: **Ed van Mourik & Neil Jordan**

Thanolelo mo Setswaneng e dirilwe ke: **Boitshepho Modise.**

Translated into Setswana by: **Boitshepho Modise.**

O ka segolola le go dirisa setshwantsho se se fa tlase.

Cut out and use the below eye template.

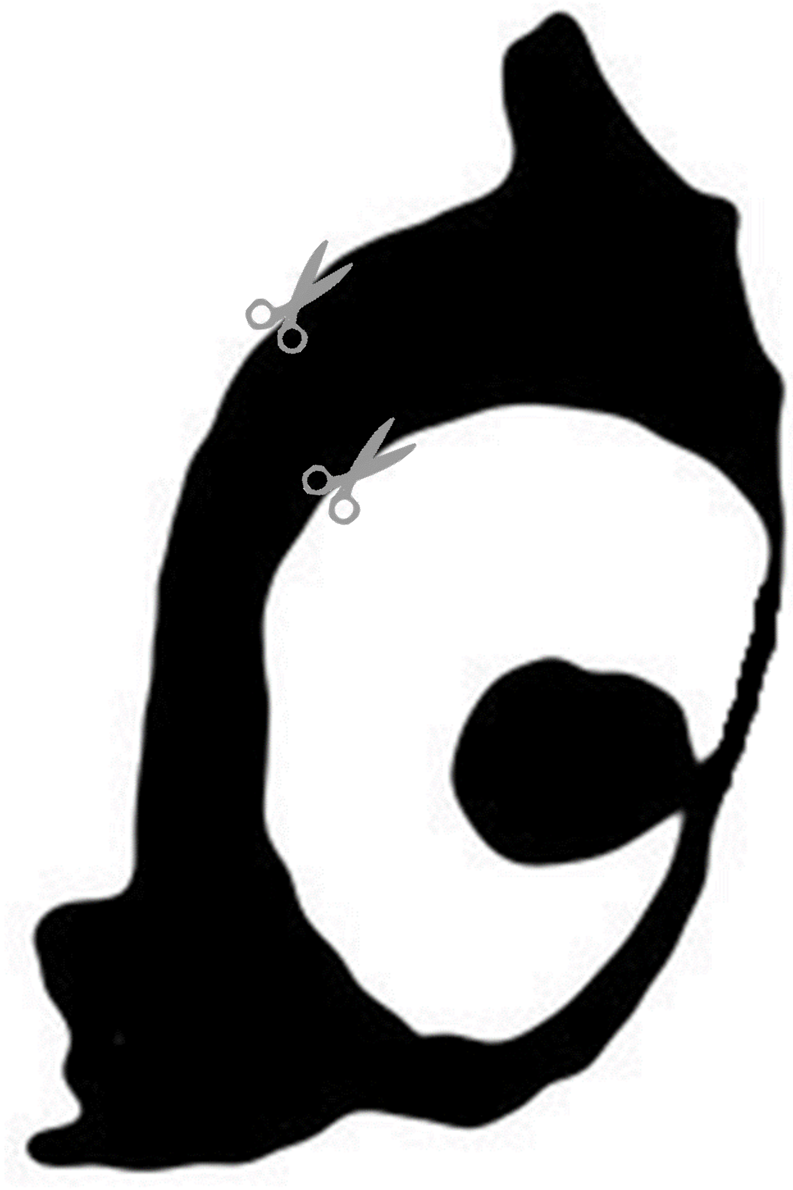

Supplement: Supplementary file 2 — Supplementary Information [file 42003_2020_1156_MOESM2_ESM.pdf]
