# Supplementary material for: Artificial eyespots on cattle reduce predation by large carnivores
Source: Commun Biol. 2020 Aug 7;3:430. doi: 10.1038/s42003-020-01156-0 (PMC7414152; doi:10.1038/s42003-020-01156-0)
Supplement: Supplementary file 5 — Reporting Summary [file 42003_2020_1156_MOESM5_ESM.pdf]

nature research

Corresponding author(s): Neil Jordan
Last updated by author(s): May 26, 2020

Reporting Summary

Nature Research wishes to improve the reproducibility of the work that we publish. This form provides structure for consistency and transparency in reporting. For further information on Nature Research policies, see Authors & Referees and the Editorial Policy Checklist.

Statistics

For all statistical analyses, confirm that the following items are present in the figure legend, table legend, main text, or Methods section.

n/a	Confirmed	
☐	☒	The exact sample size (n) for each experimental group/condition, given as a discrete number and unit of measurement
☐	☒	A statement on whether measurements were taken from distinct samples or whether the same sample was measured repeatedly
☐	☒	The statistical test(s) used AND whether they are one- or two-sided<br>*Only common tests should be described solely by name; describe more complex techniques in the Methods section.*
☒	☐	A description of all covariates tested
☐	☒	A description of any assumptions or corrections, such as tests of normality and adjustment for multiple comparisons
☐	☒	A full description of the statistical parameters including central tendency (e.g. means) or other basic estimates (e.g. regression coefficient) AND variation (e.g. standard deviation) or associated estimates of uncertainty (e.g. confidence intervals)
☐	☒	For null hypothesis testing, the test statistic (e.g. F, t, r) with confidence intervals, effect sizes, degrees of freedom and P value noted<br>*Give P values as exact values whenever suitable.*
☒	☐	For Bayesian analysis, information on the choice of priors and Markov chain Monte Carlo settings
☒	☐	For hierarchical and complex designs, identification of the appropriate level for tests and full reporting of outcomes
☒	☐	Estimates of effect sizes (e.g. Cohen's d, Pearson's r), indicating how they were calculated

Our web collection on statistics for biologists contains articles on many of the points above.

Software and code

Policy information about availability of computer code

Data collection	Data were entered using Microsoft Excel and Microsoft Access - Microsoft Office 365 Pro Plus.
Data analysis	All statistical analyses were performed in the program R (Version 3.5.2) and RStudio (Version 1.1.463, downloaded 15/01/2018)

For manuscripts utilizing custom algorithms or software that are central to the research but not yet described in published literature, software must be made available to editors/reviewers. We strongly encourage code deposition in a community repository (e.g. GitHub). See the Nature Research guidelines for submitting code & software for further information.

Data

Policy information about availability of data

All manuscripts must include a data availability statement. This statement should provide the following information, where applicable:

- Accession codes, unique identifiers, or web links for publicly available datasets
- A list of figures that have associated raw data
- A description of any restrictions on data availability

Data availability
The datasets generated during and/or analysed during the current study are available in the Zenodo repository, [https://doi.org/10.5281/zenodo.3877999]80.
All figures have associated raw data. There are no restrictions imposed on data availability.

Field-specific reporting

Please select the one below that is the best fit for your research. If you are not sure, read the appropriate sections before making your selection.

☐ Life sciences ☐ Behavioural & social sciences ☒ Ecological, evolutionary & environmental sciences

For a reference copy of the document with all sections, see nature.com/documents/nr-reporting-summary-flat.pdf

Ecological, evolutionary & environmental sciences study design

All studies must disclose on these points even when the disclosure is negative.

Study description

To test whether attacks on free-ranging livestock by wild large predators could be prevented by painting artificial eyespots on cattle, we selected 14 cattle-posts (each with one cattle herd) that had reported high predation in recent months. Within each herd, adult cattle were assigned into one of three treatment groups: 1- artificial eyespots; 2- cross-marked; or 3- unmarked. During the study we undertook 49 painting sessions before the cattle were released from overnight fenced enclosures (interval between painting sessions mean=29.61 days, sd=14.33). We applied all three treatments during each painting session, and noted herd composition and predation events that had occurred since our last visit. We also recorded exposure to risk using cattle movement data.

Research sample

Fourteen (14) cattle herds (6 - 110 head of cattle in each) were selected. This was a good representation of cattle herds exposed to predation risk within the study area. The cattle breed was Tswana of the Sanga group (Bos taurus africanus). Identification features such as existing tag ID, coat colour, sex, age and distinguishing features such as horns, were recorded for individual cattle. Total number of cattle treatments throughout the study area was 1992. Local free-ranging large predator guild included lion (Panthera leo), leopard (P. pardus), spotted hyena (Crocuta crocuta), African wild dog (Lycaon pictus), and cheetah (Acinonyx jubatus).

Sampling strategy

During 2015 and 2016, paired artificial eyespots were painted on the rumps of members of each herd after being herded into a cattle crush during the first few hours post-sunrise before cattle were released for the day. A procedural control for the effect of paint and processing (a painted cross-mark) was introduced during the 2017-2018 study period. During this time approximately one-third of each herd was painted with the artificial eyespots and one third with the control cross-mark. The rest of the herd (approximately one-third) was handled in the crush but left unmarked. Depredation event samples occurred in situ.

Data collection

Neil Jordan and Cameron Radford collected data with field assistance. Data was recorded on data sheets and later entered into Microsoft Access and Microsoft Excel for storage. Cattle and treatment data was collected during the processing of cattle at each cattlepost after sunrise. Depredation data was collected during depredation investigations as they occurred.

Timing and spatial scale

May 2015 and December 2018. Throughout the experiment, paint would typically begin to wear off after after 24 days, therefore paint was replaced cyclically approximately every four weeks during the study period (interval between painting visits mean=29.61 days, sd=14.33). Herds were excluded from the study after 24 days if they had not had their paint reapplied within this time period. If a herd was not re-painted within this time frame, then the herd was randomly re-painted on the next visit. Study herds were not processed during the rainy season months of December to February due to logistical constraints of researchers accessing the cattleposts. Study area approximately 400 square kilometers.

Data exclusions

Calf and juvenile cattle were not selected for treatment exposure. This was because these age groups had different exposure to predation (calves were kept in enclosures and juveniles for some periods as well). Also, calves and juveniles were not processed with adults through the crush for treatment application. This was pre-established before the study.

Reproducibility

We conducted one large field experiment. All code and a detailed description of the techniques used have been provided to allow the study to be reproduced.

Randomization

Treatments for individual cattle were haphazardly selected during the procession that cattle entered the crush to exit the overnight enclosure.

Blinding

Blinding was not relevant to the study.

Did the study involve field work? ☒ Yes ☐ No

Field work, collection and transport

Field conditions

Average temperature ranges were 6 to 25 degrees celcius in the coldest month (June) and 18.5 to 35 degrees in the hottest month (October). Rainy season is December to March with a peak average rainfall in January of 104mm. An Early flood season occurs in the Okavango Delta from April to July, and a Late Flood season from August to November.

Location

Study boundaries: -19.602775 23.543952, -19.597340 23.932884, -19.991444 23.948719, -19.918842 23.592166, -19.747516 23.416596. Approximately 945m elevation. Flat terrain.

Access and import/export

Sites were accessed by vehicle, keeping to established tracks as much as possible. Experimental procedures were carried out on foot. The study was undertaken with the support of the Department of Wildlife and National Parks (DWNP), and field work was conducted under the Botswana Predator Conservation Trust's (BPCT) long-running large predator research programme in northern Botswana (Research Permit EWT 8/36/4 XXXVIII (14).

Disturbance	The study did not cause any disturbance.

Reporting for specific materials, systems and methods

We require information from authors about some types of materials, experimental systems and methods used in many studies. Here, indicate whether each material, system or method listed is relevant to your study. If you are not sure if a list item applies to your research, read the appropriate section before selecting a response.

Materials & experimental systems

n/a	Involved in the study
☒	☐ Antibodies
☒	☐ Eukaryotic cell lines
☒	☐ Palaeontology
☐	☒ Animals and other organisms
☒	☐ Human research participants
☒	☐ Clinical data

Methods

n/a	Involved in the study
☒	☐ ChIP-seq
☒	☐ Flow cytometry
☒	☐ MRI-based neuroimaging

Animals and other organisms

Policy information about studies involving animals; ARRIVE guidelines recommended for reporting animal research

Laboratory animals	NA
Wild animals	Local free-ranging large predator guild included lion (Panthera leo), leopard (P. pardus), spotted hyaena (Crocuta crocuta), African wild dog (Lycaon pictus), and cheetah (Acinonyx jubatus). None of these species were captured for the study.
Field-collected samples	NA
Ethics oversight	Ethics approval (17/51A) from the Animal Ethics Committee of the University of New South Wales, Australia.

Note that full information on the approval of the study protocol must also be provided in the manuscript.
